# Mortuary Report for March

**Published:** 1880-04

**Authors:** 


### MARCH REPORT OF DEATHS AND INTERMENTS IN BALTIMORE.

| CAUSES OF DEATH. | NO. | CAUSES OF DEATH. | NO. |
|---|---|---|---|
| Abscess, Parotid Gland | 1 | Gastro Enteritis | 1 |
| Albumenuria | 2 | Hernia (Strangulated) | 1 |
| Apoplexy | 7 | Hæmorrhage of Brain | 3 |
| Ascites | 2 | " Lungs | 3 |
| Asphyxia | 1 | " Umbilical | 2 |
| Asthenia | 5 | Hæmoptysis | 1 |
| At Labor | 1 | Heart Clot | 1 |
| Bright's Disease | 6 | Hydrocephalus | 5 |
| Burns | 5 | Hydrothorax | 1 |
| Capillary Bronchitis | 11 | Inanition | 10 |
| Cancer, Parotid Gland | 1 | Insanity | 1 |
| " Breast | 2 | Inflammation of Bowels | 2 |
| " Glands Neck | 1 | " Stomach | 4 |
| " Bowels | 1 | " Bronchi | 6 |
| " Uterus | 3 | " Larynx | 2 |
| " Stomach | 2 | Infantile Catarrh | 3 |
| Casualties | 1 | Inflammation of Pericardium | 1 |
| Cirrhosis of Liver | 2 | " Peritoneum | 1 |
| Cynanche Tonsillaris | 1 | " Pleura | 1 |
| Congestion of Brain | 5 | Intussusception | 1 |
| " Lungs | 5 | Intemperance | 1 |
| Consumption of Bowels | 1 | Jaundice | 1 |
| " Lungs | 104 | Malformation | 2 |
| Convulsions | 17 | Marasmus | 19 |
| " Puerperal | 1 | Measles | 1 |
| Croup | 19 | Meningitis | 3 |
| Catarrh, Stomach | 1 | " Tubercular | 10 |
| Dentition | 1 | " Cerebro Spinal | 1 |
| Dyspepsia | 1 | Mal Nutrition | 2 |
| Diabetes | 1 | Murder | 1 |
| Diarrhœa | 2 | Necrosis Lary'al Cartilage | 1 |
| Diphtheria | 24 | Old Age | 20 |
| Disease of Brain | 3 | Paralysis | 14 |
| " Heart | 15 | Pneumonia | 46 |
| Dropsy (Gen'l) | 4 | " Typhoid | 3 |
| Drowned | 3 | " Pleuro | 3 |
| Dysentery | 1 | " Broncho | 1 |
| Epilepsy | 1 | Phlegmasia Dolens | 1 |
| Erysipelas | 1 | Premature Birth | 3 |
| Embolism | 1 | Protracted Labor | 1 |
| Fever, Catarrh | 1 | Pyæmia | 1 |
| " Puerperal | 4 | Rheumatism, Inflammatory | 2 |
| " Remittent | 2 | Rupture, Uterus | 1 |
| " Scarlet | 22 | Scrofula | 2 |
| " Typhoid | 7 | Softening of Brain | 1 |
| Gangrene (Senile) | 1 | Stricture, Intestines | 2 |

### MORTUARY REPORT—(Continued).

| CAUSES OF DEATH. | NO. | CAUSES OF DEATH. | NO. |
|---|---|---|---|
| Stricture, Oesophagus | 1 | Nervous Prostration | 1 |
| Suppression, Urino | 1 | Neurasthenia | 1 |
| Suicide | 2 | Lesion of the Meninges | 1 |
| Syphilis | 2 | Irrepressible Vomiting | 1 |
| Tabes Mesenterica | 1 | | |
| Trismus Nascentium | 5 | Total | 528 |
| Ulcer of Stomach | 1 | | |
| Uræmia | 2 | Annual Death Rate per 1000 during the month | 16.08 |
| Unknown, Infantile | 6 | | |
| Whooping Cough | 4 | | |
| Wounds | 3 | Estimated Population | 393,796 |

### THE DEATHS REGISTERED IN THE MONTH INCLUDE

| | | | |
|---|---|---|---|
| Under 1 year | 121 | Between 30 and 40 years | 42 |
| Between 1 and 2 years | 42 | " 40 and 50 " | 35 |
| " 2 and 5 " | 49 | " 50 and 60 " | 45 |
| | | " 60 and 70 " | 29 |
| Under 5 years | 212 | " 70 and 80 " | 36 |
| Between 5 and 10 years | 29 | " 80 and 90 " | 18 |
| " 10 and 15 " | 10 | " 90 and 100 " | 0 |
| " 15 and 20 " | 14 | " 100 and 110 " | 1 |
| " 20 and 30 " | 57 | Above 110 years | |

### NATIVITY, ETC.

| | |
|---|---|
| United States (White) | 276 |
| " (Colored) | 151 |
| Foreign | 101 |
| Total | 528 |
| Corresponding month 1879 | 716 |
| Still Birth | 59 |

By Order: A. R. Carter, Secretary. James A. Steuart, M. D., Commissioner of Health and Registrar.

